# Automatic activation of the shared-digit network in the solution of complex multiplication problems

**DOI:** 10.3758/s13421-025-01766-1

**Published:** 2025-08-15

**Authors:** Smadar Sapir-Yogev, Sarit Ashkenazi

**Affiliations:** The Seymour Fox School of Education, The Hebrew University of Jerusalem, 91905 Jerusalem, Israel

**Keywords:** Multiplication table, Complex multiplication, Associative network, Arithmetic, Mathematical knowledge, Numerical cognition

## Abstract

Previous studies have shown that the associative network of single-digit multiplication problems is automatically activated, even when participants perform irrelevant tasks, and that single-digit multiplication problems automatically activate all single-digit problems sharing at least one digit with them (the shared-digit network; SDN). We examined whether the SDN would also be automatically activated when participants perform an irrelevant task. Specifically, we asked whether complex multiplication problems (e.g., 2 × 12 = ) automatically activate all single-digit problems that share digits with them. In Experiment 1, participants solved all complex problems whose solutions were less than 100. In Experiments 2 and 3, participants solved sets of complex problems that differed in SDN size and in carryover status and were matched in problem size. Participants reported the strategies they used to solve the problems. Results showed that SDN size, which reflected the number of single-digit problems sharing digits with the complex problem, predicted speed and accuracy in the solution of complex problems. Regardless of carryover status and strategy, participants solved complex problems with small SDNs more quickly than complex problems with large SDNs. Regardless of carryover status, participants used retrieval more often when solving problems with a small SDN than with a large SDN. Thus, we have demonstrated that SDN size determines speed and accuracy in the solution of complex multiplication problems, and that the SDN is automatically activated even during performance of an irrelevant task.

**Supplementary Information:**

The online version contains supplementary material available at 10.3758/s13421-025-01766-1.

## Introduction

Complex multiplication problems, such as 3 × 17 = 51, are used in a variety of everyday situations. For example, if a mother wants to buy falafel for herself and her two children, and each pita bread filled with this delicacy costs 17 Shekels, she has to solve the problem 3 × 17 = to find the total expense. Complex multiplication problems such as 3 × 17 = 51 are introduced and practiced in elementary school (Heirdsfield et al., 1999; LeFevre et al., 1993; Liu, 2013). However, adults solve some complex multiplication problems more quickly and more accurately than others (Liu, 2013; Masse & Lemaire, 2001). In a previous study (Sapir-Yogev et al., 2024) we demonstrated that a single-digit multiplication problem automatically activates all single-digit multiplication problems sharing at least one digit with it. These activated problems interfere with the solution of the target problem. The present study attempts to examine whether a 1 × 2-digit (hereafter, complex) multiplication problem automatically activates all *single-digit* multiplication problems sharing at least one digit with it, thus interfering with its solution as well.

Single-digit and complex multiplication problems share the problem size effect. This effect refers to the observation that single-digit problems with smaller solutions are solved more quickly and more accurately than problems with larger solutions (e.g., Campbell, 1994). Lemaire and Brun (2016) had participants verify complex multiplication problems. Participants verified small problems, whose mean solution size was 163, more quickly than large problems, whose mean solution size was 387. van der Ven et al. (2015) analyzed data from about 27,000 participants who used a computerized practice program to solve hundreds of complex multiplication problems. They found that larger problem size made a problem significantly more difficult to solve. Similarly, Liu (2013) asked elementary teaching candidates to mentally calculate the solutions to 2 × 2 digits multiplication problems. As problem size increased, both RTs and error rates increased.

Multiplication-irrelevant tasks may trigger automatic, unintentional processing of multiplication-related numerical information (Galfano et al., 2009). For example, Keha et al. (2024) asked participants to name the color with which single-digit multiplication problems were presented. Participants named the color of large problems more slowly than of small problems, and of incorrect problems that respected parity rules more slowly than of incorrect problems that violated parity rules. These results indicated that size and parity information were automatically processed even when they were not needed for the task. The associative links between the operands and the solution of multiplication problems (e.g., Campbell & Graham, 1985; Siegler, 1988) may also be activated by automatic processes that do not require intentional processing (e.g., Zbrodoff & Logan, 1986) and that do not involve multiplication. The associative confusion effect (Winkelman & Schmidt, 1974) exemplifies such processes. Participants reject incorrect addition problems whose solution is the product of its addends (e.g., 4 + 5 = 20) more slowly than similar problems with no confusion (e.g., 4 + 5 = 15). The number-matching task is another example of the automatic activation of these associative links. In this task, participants are presented with cues (e.g., 7 and 6) followed by a probe (e.g., 42), and are asked to decide whether the probe is identical to any of the cues. Galfano et al. (2009) showed that when the probe was the product of the cues (e.g., 42), participants were slower to reject it than an unrelated probe (e.g., 45). Using the same paradigm with complex multiplication, Tronsky (2016) let undergraduates practice complex problems (e.g., 4 × 17). A week later they were presented with two cues (e.g., 4 and 17) followed by a probe (e.g., 68) and were asked to decide whether the probe matched one of the cues. Participants showed slower response times and higher error rates for probes that were products of the cues compared to probes that were unrelated to the cues.

Single-digit and complex multiplication problems automatically activate other problems sharing digits with them (e.g., De Visscher & Noël, 2014; Galfano et al., 2003). Campbell and Graham (1985) let participants orally solve all 2–9 single-digit multiplication problems and found that most errors were correct solutions to problems that shared an operand with the presented problem. Rotem and Henik (2015) let participants verify all 2–9 single-digit multiplication problems. Half of the incorrect solutions were multiples of one of the operands and the other solutions were not related to any of the operands. The authors documented longer verification times when incorrect solutions were multiples of one of the operands, relative to other incorrect solutions. Domahs et al. (2006) reanalyzed Campbell’s (1997) data, which included the oral solution of all 2–9 single-digit problems by adults, and found that most of the incorrect solutions shared the tens digit with the correct solution. Domahs et al. (2007) let participants verify single-digit multiplication problems. The tens digit in half of the incorrect solutions matched the tens digit of the correct solutions (“consistent”), while in the other half the presented and correct tens digits did not match (“inconsistent”). Incorrect problems with consistent solutions were harder to reject than incorrect problems with inconsistent solutions. Tronsky (2016) had students study complex problems, and then presented them with the same problems in a verification task. The suggested incorrect solutions were either a close multiple of the operands, sharing the tens digit with the correct solutions (e.g., 4 × 14 = 52), a close multiple of the operands, but not sharing the tens digit with the correct solution (e.g., 4 × 14 = 60), or an incorrect solution which was not a multiple of any of the first two numbers. Close multiples sharing the single-digit operand were rejected more slowly and less accurately than unrelated incorrect solutions. Close multiples sharing the tens digit in the solution with the correct solution were rejected less accurately than close multiples not sharing a tens digit with the correct solution.

In line with the evidence for automatic activation and interference between problems sharing digits, we have shown that a single-digit multiplication problem automatically activates all other 2–9 single-digit multiplication problems sharing digits with it (Sapir-Yogev et al., 2024). These activated problems are the Shared-Digit Network (SDN) of the target problem. SDN size is the number of problems in a target problem’s SDN. We presented participants with all single-digit multiplication problems in verification and retrieval tasks, and found that SDN size predicted variance in speed and accuracy beyond problem size. Moreover, participants verified and solved sets of problems which were matched in problem size and did not include five or tie problems (e.g., 5 × 3, 3 × 3), that are easier to solve (De Brauwer et al., 2006; Graham & Campbell, 1992). Problems with a small SDN were solved faster than problems with a large SDN (Sapir-Yogev et al., 2024). We concluded that the problems in the SDN of a target problem interfere with the solution of that problem. The larger the SDN, the slower and less accurate the solution. Single-digit problems with a small SDN are solved more quickly than problems with a large SDN, because they are subjected to less interference. The SDN conceptualization allows for the definition of part SDNs. An SDN-by-operands would consist of all problems sharing digits with the digits in the operands of the target problem. An SDN-by-solution would consist of all problems sharing digits with the digits in the solution of the target problem.

**Fig. 1:**
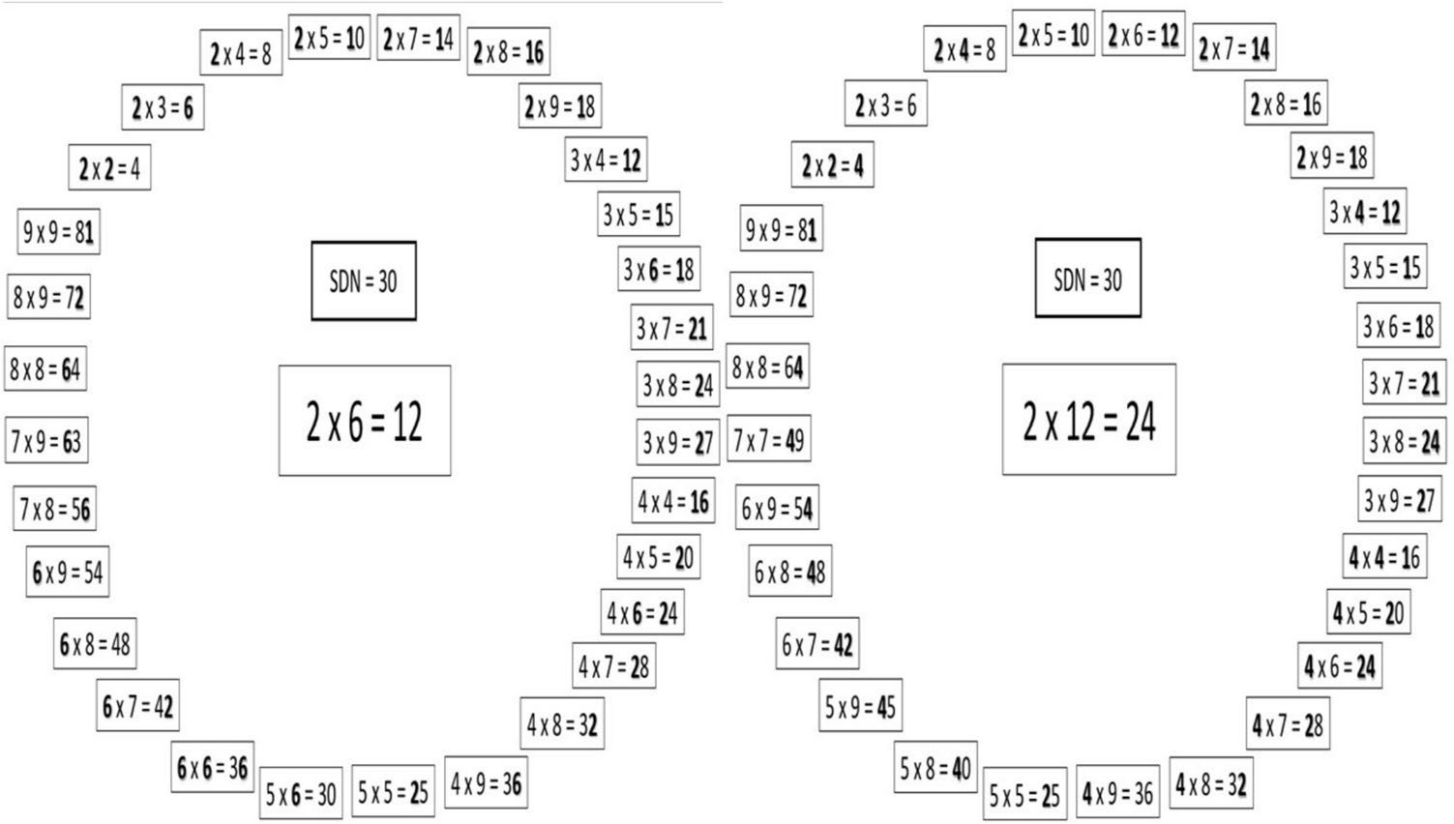
The SDNs of the problems 2 × 6 = 12 (left) and 2 × 12 = 24 (right)

In this study, we examine whether a network of single digit multiplication problems is automatically activated when participants solve *complex* multiplication problems. Mastery of single-digit problems facilitates a fast leap in complex multiplication ability, indicating that the networks of single-digit and complex multiplication may be connected (Hickendorff et al., 2019; Linsen et al., 2016; van der Ven et al., 2015). However, no previous study has systematically examined the connections between single-digit and complex multiplication problems. We define the SDN of a complex multiplication problem as consisting of all *single-digit* problems sharing at least one digit with the digits in the complex problem. Figure 1 shows the SDN of the single-digit problem 2 × 6 = 12 (on the left) and the SDN of the complex problem 2 × 12 = 24 (on the right). Note that while the target problem may be single-digit or complex, both SDNs are composed of *single-digit* problems. As shown in Fig. 1, some problems share digits only with the operands of the target problem, only with the solution, or with both the operands and the solution. For example, the problem 9 × 9 = 81 shares the digit 1 with the operand 12 of the target problem 2 × 12 = 24. The problem 6 × 9 = 54 shares the digit 4 with the solution 24 of that target problem, and the problem 2 × 7 = 14 shares the digits 2, 1, and 4 with the operands and the solution of that target problem. We hypothesize that the digits in a complex problem would automatically activate all *single-digit* problems that contain them, even when the task at hand is to solve a complex, rather than a single-digit, problem. We hypothesize that this automatic activation of the network of single digit problems will interfere with the solution of complex problems. Thus, we hypothesize that SDN size will explain differences in speed and accuracy in the solution of complex multiplication problems.

In Experiment 1, we will examine the hypothesis that SDN size will predict speed and accuracy in the solution of all complex multiplication problems with solutions smaller than 100, beyond problem size. In Experiments 2 and 3, we will examine the hypothesis that complex problems with a small SDN will be solved more quickly than complex problems with a large SDN using selected groups of problems.

## Experiment 1: Solution of a diverse set of complex multiplication problems

Experiment 1 examines the hypothesis that SDN size will predict speed and accuracy in the solution of complex multiplication problems beyond problem size, by having participants solve a large set of complex problems, with a wide range of SDN sizes.

### Method

#### Participants

The sample included 32 undergraduates of an Israeli university, 77% of them women, ranging in age from 20 to 26 years (mean age = 22.63 years, *SD* = 1.56), who received credit for their participation. Participants’ mean number of years of education was 12.72 (*SD* =.79). All participants were born in Israel and spoke Hebrew as their native language, were right-handed, and reported having no learning disabilities. Sample-size for hierarchical regression analyses with two predictors (problem size and SDN size) was conducted before data collection, was calculated with the G*Power 3.1 software (Faul et al., 2009), based on an effect size of *f* ∼.35 and a power of 1 − ß > 0.80, and indicated a sample size of 31. The study received Institutional Review Board ethics approval.

#### Stimuli

The stimuli consisted of all multiplication problems having one single and one double-digit operand, whose solutions were less than 100 (i.e., all complex problems with five digits (including the digits in the solution). Each problem was presented once, with the single digit operand first (e.g., 3 × 17 =), for a total of 106 problems (see Appendix 1 for the list of problems). Problems were displayed in a pseudorandom order. Problems with the same operand did not appear consecutively, and problems with the same solution appeared with at least four other problems between them.

#### Procedure

The task was programmed with the Gorilla Experiment Builder for online studies (Gorilla.sc; Anwyl-Irvine et al., 2020). Participants were asked to sit in a quiet room with no distractions, to use a computer rather than a phone or a tablet, and to turn off their phone. Each trial began with a fixation point of 500 ms, after which the problem was presented. The problems for all three Experiments in this article were presented in black Arial font, and were.5 cm in size on a 15.5 × 34.5–cm screen. The problems were presented 5.5 cm from the top and 12.5 cm from the left of the screen (see Fig. S1 in the Supplementary Material for a sequence of screen presentations). Participants were asked to solve the problems as quickly and as accurately as possible, by typing the result on the keyboard, and then pressing the Enter key. If participants did not respond within 13 s, they were prompted to do so. To familiarize participants with the task, the experiment began with 24 single-digit multiplication training problems. The software recorded RT and accuracy rate.

### Results

The accuracy of one participant was more than two standard deviations below the mean of the entire sample, and their responses were omitted from all further analyses.

To examine whether SDN size explains variance in the solution of multiplication problems beyond problem size, we conducted two hierarchical regression analyses, one for RT and one for accuracy. Problem size was entered in Step 1, and SDN size was entered in Step 2. Table 1 shows that problem size explained 9.4% of the variance in RT. Most importantly, SDN size explained an additional 36.9% of the variance in RT beyond problem size. Similarly, problem size explained 14.4% of the variance in accuracy, and SDN size explained an additional 12.8% of the variance in accuracy beyond problem size.

**Table 1:** Linear regressions predicting RT and accuracy rate in Experiment 1

Dep. var	Step	Indep. var	*B*	*SE*	β	*t*	*p*	∆*R*^2^
RT	Step 1	Problem size	22.35	6.81	.31	3.28	.001	.094
	Step 2	Problem size	21.85	5.27	.30	4.15	.000	
		SDN size	200.06	23.81	.61	8.40	.000	.369
Acc	Step 1	Problem size	−.17	.04	−.38	−4.18	.000	.144
	Step 2	Problem size	−.17	.04	−.37	−4.46	.000	
		SDN size	−.73	.17	−.36	−4.26	.000	.128

Since multiples of 10 and 11 may be solved by rules (n × 10 = n0; n × 11 = nn), we conducted two additional hierarchical regression analyses, one for RT and one for accuracy with a subset of 90 problems, excluding problems that are multiples of 10 and 11. Problem size was entered in Step 1 and SDN size was entered in Step 2 (see Table S1 in the Supplementary Material). The results did not change. Problem size explained 7.9% of the variance in RT (β =.28, *p* =.007). SDN size explained an additional 13.7% of the variance in RT beyond problem size (β =.37, *p* <.001). Similarly, problem size explained 14.1% of the variance in accuracy (β = −.38, *p* <.001), and SDN size explained an additional 4.9% of the variance in accuracy beyond problem size (β = −.22, *p* =.023). These analyses show that the effect of SDN on performance is not due to the use of rules.

It may be argued that since the participants were presented only with the operands, the entire SDN effect may be due to the SDN of the operands. This SDN would consist of all single-digit problems sharing digits with the operands of a complex problem. To examine this argument, we calculated for each problem the size of the SDN-by-operands and the size of the SDN-by-solution, which is the number of single-digit problems sharing digits with the solution.

We conducted two hierarchical regression analyses with all problems, one for RT and one for accuracy. Problem size was entered in Step 1, SDN-by-operands size was entered in Step 2 and SDN-by-solution size was entered in Step 3. Table S2 shows that problem size explained 9.4% of the variance in RT. SDN-by-operands size explained an additional 23.1% of the variance in RT beyond problem size, and SDN-by-solution explained an additional 13% of the variance in RT beyond problem size and SDN-by-operand size. Similarly, problem size explained 14.4% of the variance in accuracy, SDN-by-operands size explained an additional 8.7% of the variance in accuracy beyond problem size, and SDN-by-solution explained an additional 3% of the variance in accuracy beyond problem size and SDN-by-operands size.

In addition to the effect of problem size, our data reaffirms the five-effect in complex multiplication. Single-digit and complex problems that contain a five operand are easier to solve than other problems (e.g., De Brauwer et al., 2006; Hinault et al., 2015; Masse & Lemaire, 2001). Table S3, and the supplemental analysis following it, show that in our data too, five problems are solved more quickly and more accurately than nonfive problems.

### Discussion

In Experiment 1, the participants solved all complex multiplication problems whose solutions were smaller than 100. SDN size predicted both RT and accuracy in the solutions of these problems beyond problem size, even after we removed the multiples of ten and eleven, which may be solved by rules. Furthermore, both the SDN-by-operands and the SDN-by-solution predicted unique variance in RT and accuracy beyond problem size. Since the SDN-by-operands consists of all single-digit problems sharing digits with the operands of the complex problem, and the SDN-by-solution consists of all single-digit problems sharing digits with the solution of the complex problem, these results show that the SDN effect is not caused by the digits in the operands alone but rather by all digits in the problem.

Our results reveal a new similarity between single-digit and complex multiplication. In both sets of problems, SDN size determines speed and accuracy in the solution. This highlights the involvement of single-digit problems in complex multiplication (Hickendorff et al., 2019; Linsen et al., 2016; van der Ven et al., 2015). All digits in a complex problem, in the operands and in the solution, automatically activate all single-digit problems sharing them, and these activated problems interfere with the solution of the target problem. The larger interference in problems with a larger SDN slows speed and lowers accuracy in the solution of these problems relative to problems with a smaller SDN.

Using tasks that did not involve single-digit multiplication, previous studies documented an automatic activation of the links between the operands and the correct solution (Galfano et al., 2009; Winkelman & Schmidt, 1974; Zbrodoff & Logan, 1986), and between the operands and their close multiples (Galfano et al., 2003) in single-digit problems. Solving complex problems involves the retrieval of single-digit problems as well as the performance of other operations (Tronsky, 2005). However, the algorithm for the solution of a problem such as 3 × 17 = 51 does not necessitate the retrieval of all single-digit problems containing the digit 5. Yet the results of Experiment 1 show that such problems are automatically activated during the solution of this problem. Thus, the current experiment demonstrates the automatic activation of problems that are not needed for the task at hand, and is the first to show the obligatory activation of a *single-digit* network in complex multiplication tasks.

Using a nonmultiplication task (the number matching task), Tronsky (2016) showed an automatic activation of the solutions of complex multiplication problems by their operands. In contrast with our study, the automatic activation in Tronsky’s (2016) study occurred between the operands and the solution of the *complex* problem itself. We showed an automatic activation of *single-digit* problems by the digits in the complex problem. The single-digit network is well-rehearsed (Campbell, 1987); thus, this automatic activation could occur without practicing the complex problems. Automatization without practice was not expected in Tronsky’s (2016) study, because he was interested in the links between the operands and solution of the complex problems, which are rehearsed less than single-digit problems (Schulz, 2018).

Experiment 1 showed that all digits in the complex problem give rise to the SDN effect. Some complex problems may be solved by retrieval (Tronsky, 2016). In these problems, it is easy to see how the digits in the solution, which may be part of the representation of the problem (Campbell & Oliphant, 1992), might participate in the activation of the SDN. Other complex problems are solved mostly by calculation (van der Ven et al, 2015). In such problems, the digits in the operands may activate all single-digit problems that share them. A horse race between calculation and retrieval may then ensue (Ashcraft, 1987; Baroody, 2018; Logan, 1988), during which the digits in the solution are accessed and activate the remaining problems in the SDN.

Finally, the results of Experiment 1 provide more evidence for the existence of the problem size effect (Liu, 2013; van der Ven et al., 2015) and the five effect in complex multiplication. The five effect refers to the observation that problems that contain a five operand are easier to solve than other problems (e.g., De Brauwer et al., 2006). Masse and Lemaire (2001) as well as Hinault et al. (2015) asked adults to verify complex multiplication problems. They documented shorter RTs and greater accuracy when the single-digit operand was five than when it was not five. Previous explanations for the five effect included higher frequencies of five problems in textbooks (Ashcraft, 1987; Siegler, 1988), a separate storage for five problems, leading to reduced interference (Campbell & Oliphant, 1992), and less proactive interference to five problems (De Visscher & Noël, 2014). These explanations rely on a specific learning sequence, are confounded with problem size (De Visscher & Noël, 2014), and have not found much empirical support (Verguts & Fias, 2005). Our explanation does not suffer from such drawbacks. We suggest that complex five problems are easier to solve than nonfive problems because they share digits with fewer single-digit problems, their SDNs are smaller, and, consequently, they receive less interference from other problems.

The SDN effect was originally found in single-digit problems, which are solved predominantly by retrieval (Siegler, 1988). The solution of complex problems may involve carryover. Carryover (also known as regrouping or carrying) refers to a process where a digit is moved from one place value column to the next when a product is greater than nine. While many noncarry complex problems may be solved by retrieval (Tronsky, 2016), carryover complex problems are more calculated (Fürst & Hitch, 2000). In Experiment 1, we used a large group of complex problems, regardless of carryover status. In Experiment 2, we created equal groups of problems, with and without carryover, matched in problem size. This enabled us to see whether the SDN effect would recur not only in noncarry problems but also in carryover, calculated problems.

## Experiment 2: Solution of equal sets of size-matched problems differing in SDN size and carry status

Experiment 1 showed that SDN size predicted speed and accuracy in the solution of all complex multiplication problems with solutions smaller than 100, beyond problem size. In Experiment 2, we aim to see whether the effect of SDN size occurs in selected sets with and without carryover.

The SDN effect was found in single-digit problems (Sapir-Yogev et al., 2024) that are mostly solved by retrieval (Campbell & Oliphant, 1992). Some complex noncarry problems may be well-rehearsed (e.g., 2 × 12 = 24, 2 × 21 = 42), and may be solved mostly by retrieval as well (Tronsky, 2016). Carryover problems are usually harder to solve than noncarry problems, since they require an extra calculation step, and hence more working memory resources relative to noncarry problems (Imbo et al., 2007a, Imbo et al., 2007b). Indeed, college and university students took longer to solve double-digit problems with carry relative to problems without carry (Ashcraft & Faust, 1994), and made more calculation errors when solving carry relative to noncarry multidigit problems (Fürst & Hitch, 2000). The existence of an SDN effect in noncarry as well as in carryover problems would show that the digits in a complex multiplication problem automatically activate the single-digit problems that contain them, regardless of the strategy used to solve the complex problems.

We examined the hypothesis that participants will solve complex problems with a smaller SDN more quickly and more accurately than complex problems with a larger SDN, regardless of carryover status, by creating four sets of problems matching in size and in the single-digit operand, but differing in SDN size and in their carry status. To eliminate the possibility that five, ten, or 11 problems are responsible for the effect, we did not include such problems in the sets.

### Method

#### Participants

The sample consisted of 32 Amazon Mechanical Turk workers, 53% of them women, ranging in age from 25 to 58 years (mean age = 33.54 years, *SD* = 8.28). Participants’ mean number of years of education was 15.69 (*SD* = 1.26). All participants were born in the USA and spoke English as their native language, were right-handed, and reported having no learning disabilities. The participants received a compensation of USD 2.5 for their participation. Sample-size for two-way repeated-measures analyses of variance (ANOVAs) for this experiment and for Experiment 3 was conducted before data collection and was calculated using G*Power 3.1 software (Faul et al., 2009), based on an effect size of *f* ∼.35 and a power of 1 − ß > 0.80, and indicated the need for 13 participants. Nevertheless, we wanted all three experiments to have similar sample sizes and to have more than 30 participants, to ensure the assumptions of normality. The study received Institutional Review Board ethics approval.

#### Stimuli

We selected four sets of problems that matched in problem size and in the single-digit operands (see Appendix 2 for the full sets). Two sets were noncarry problems differing in SDN size.^1^ None of the experimental problems were five, ten, or 11 problems. Each experimental problem appeared six times, with the small operand first, for a total of 144 problems. Problems with the same double-digit operand did not appear consecutively. Problems with the same two operands appeared with at least four other problems between them. Otherwise, presentation order was random, and problems from all four sets were interleaved.

#### Procedure

The procedure was identical to the procedure of Experiment 1. The experiment began with 24 training trials, half of them with carry. The practice problems did not contain double-digit operands or solutions that appeared in the experimental problems.

#### Results

The accuracy of two participants was more than two standard deviations below the mean of the entire sample, and their responses were omitted from all further analyses.

For each participant, we calculated the mean RT for correct responses as well as the accuracy rate for problems in either condition.

**Fig. 2:**
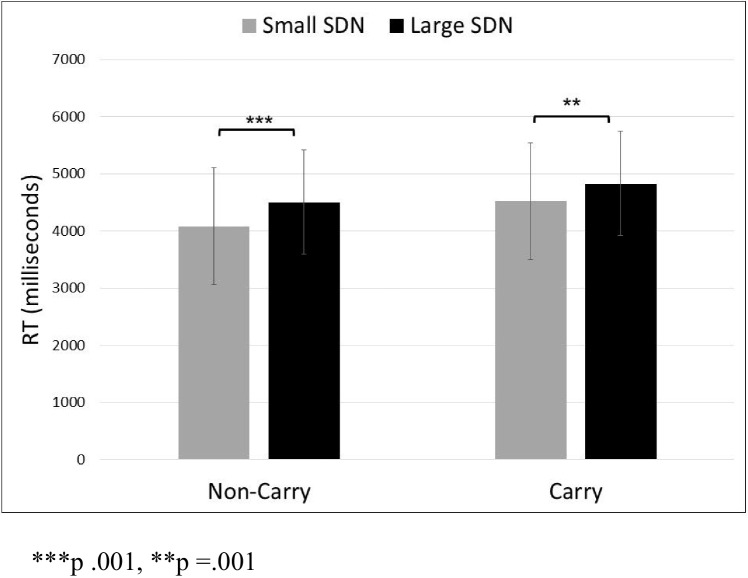
Mean RT for problems with small and large SDN size, with and without carry-over, in Experiment 2

Since our stimuli sets contained a difference in SDN size between problems with a small SDN with or without carryover, a repeated-measures ANOVA with two within-subjects variables (small/large SDN and carry/no-carry status) was not advisable. Nevertheless, we present such an analysis^2^, which shows main effects of SDN size and carry status on RT. A repeated-measures ANOVA with the sets of noncarry problems showed that participants solved problems with a small SDN faster (*M* = 4081, *SD* = 1021) than problems with a large SDN (*M* = 4503, *SD* = 909), *F*(1, 29) = 24.34, *p* <.001, *MSE* = 109,604.56, partial η^2^ =.46. A repeated-measures ANOVA with the carry problems showed that participants solved problems with a small SDN faster (*M* = 4,519, *SD* = 1,032) than problems with a large SDN (*M* = 4,830, *SD* = 1,046), *F*(1, 29) = 14.59, *p* =.001, *MSE* = 98,987.96, partial η^2^ =.33 (see Fig. 2). Accuracy rates were high, at ceiling levels (*M* = 94.32, *SD* = 6.14). A repeated-measures ANOVA with the sets of noncarry problems showed no difference in accuracy when participants solved problems with a small SDN (*M* = 94.36, *SD* = 6.08) than problems with a large SDN (*M* = 95.65, *SD* = 3.24), *F*(1, 29) = 1.25, *p* =.273, *MSE* = 20.15, partial η^2^ =.041. A repeated-measures ANOVA with the carry problems showed no accuracy differences when participants solved problems with a small SDN (*M* = 93.89, *SD* = 7.59) than problems with a large SDN (*M* = 93.42, *SD* = 6.93), *F*(1, 29) =.153, *p* =.669, *MSE* = 21.04, partial η^2^ =.005.

### Discussion

In Experiment 2, we examined the effect of SDN size on four sets of carry and noncarry problems that matched in the single-digit operands and in problem size but differed in SDN size. Participants solved noncarry problems with a small SDN faster than noncarry problems with a large SDN. The same pattern of results was found for problems with carryover. Participants solved carry problems with a small SDN faster than carry-problems with a large SDN. The SDN effect did not occur for accuracy in noncarry as well as carry problems, probably because accuracy rates were at ceiling levels. These findings strengthen the pattern of results found in Experiment 1. We argue that the SDN effect occurs because the single-digit problems in the SDN are automatically activated and interfere with the solution of the target complex problem. Complex problems with a small SDN are subjected to less interference to their solution and consequently are solved more quickly than complex problems with a large SDN.

Noncarry problems are easier to solve (Ashcraft & Faust, 1994), and some of them may be retrieved (Tronsky, 2016). The occurrence of an SDN effect in balanced sets of noncarry problems shows that complex problems have an SDN and that the single-digit network affects the solution of these problems. Carry problems are mostly calculated (Siegler, 1988; Siegler & Shipley, 1995). The occurrence of the SDN effect in carryover problems indicates that this effect occurs, regardless of the strategy applied to solve the problem. The occurrence of an SDN effect in calculated problems may not be entirely new. Some single-digit problems, such as 7 × 8 or 6 × 9 may be solved by calculation more than other single-digit problems (Hofman et al., 2018; LeFevre & Liu, 1997; Lemaire & & Siegler, 1995), yet the SDN effect occurs in all single-digit problems (Sapir-Yogev et al., 2024).

In Experiment 3, we used the same problem sets as in Experiment 2, while adding a strategy questionnaire. We wanted to know whether the pattern of results found in Experiment 2 would recur for participants who report that they solved the problems with different strategies. We hypothesized that regardless of the reported strategy, problems with a small SDN would be solved more quickly than problems with a large SDN. We further hypothesized that regardless of carry status, problems with a small SDN would be solved with retrieval more than problems with a large SDN, since they are subject to less interference.

## Experiment 3: Occurrence of the SDN effect in participants using various strategies

In Experiment 2, we have shown that the SDN size effect occurs in selected sets with and without carryover. In Experiment 3, we used the problem set of Experiment 2 with a strategy questionnaire. The addition of a strategy questionnaire enabled us to examine the hypothesis that problems with a small SDN would be solved more quickly than problems with a large SDN, regardless of the strategy participants used to solve them. Since noncarry problems require fewer calculation steps than carry problems, they are easier to solve, and are more likely to be solved by retrieval (Tronsky, 2016). Carryover problems require an extra calculation step compared with noncarry problems (Imbo et al., 2007a, Imbo et al., 2007b); thus, there is less chance that their practice in childhood, and occasional solution in adulthood, would lead to their solution by retrieval (Siegler, 1988). Thus, we expected participants to use retrieval in noncarry problems more than in carry problems. We hypothesized that regardless of carry status, problems with a small SDN would be solved with retrieval more than problems with a large SDN, since they encounter less interference.

### Method

#### Participants

The sample consisted of 35 students at an Israeli university, 80% of them women, ranging in age from 19 to 31 years (mean age = 22.88 years, *SD* = 3.07). Participants’ mean number of years of education was 12.66 (*SD* =.92). All participants were born in Israel and spoke Hebrew as their native language, were right-handed, and reported having no learning disabilities. The participants received credit for their participation. Sample size was based on the same calculation as for Experiment 2. The study received Institutional Review Board ethics approval.

#### Stimuli

The stimuli consisted of the same sets of problems as in Experiment 2 (see Appendix 2). Each experimental problem appeared six times, beginning with the small operand, for a total of 144 problems. Problems with the same double-digit operand did not appear consecutively. Problems with the same two operands were separated by at least four other problems. Otherwise, presentation order was random, and problems from all four sets were interleaved.

#### Procedure

The procedure was identical to the procedure of Experiment 2. The experiment began with 24 training trials, half of them with carry. The practice problems did not contain double-digit operands or solutions that appeared in the experimental problems.

At the end of the experiment, after participants solved all problems, they answered a strategy questionnaire about the way they solved each of the 24 problems. The suggested strategies were as follows:

1. Retrieval (remembered/knew the solution immediately, with no need for any calculation).
2. Calculation (multiplied the tens digit, then multiplied the units digit, and then added or the other way around. For example: 4 × 19 = (4 × 10) + (4 × 9) or 4 × 19 = (4 × 9) + (4 × 10)).
3. Rounding (rounded the double-digit number up or down and then subtracted or added the difference. For example: 4 × 19 = (4 × 20) – 4 or 4 × 21 = (4 × 20) + 4)).
4. Familiar Problem (relied on familiar problems. For example: 4 × 19 = (4 × 15) + (4 × 4)).
5. Algorithm (mentally carried out the standard multidigit multiplication algorithm).
6. Addition (turned the problem into an addition problem. For example: 2 × 11 = 11 + 11).

### Results

The accuracy of two participants exceeded two standard deviations below the mean of the entire sample, and their responses were omitted from all further analyses. The age of two participants was more than two standard deviations above the mean of the entire sample, and their responses were also omitted from the analyses. One more participant did not answer the questionnaire, and one other participant stopped working after the break. The data of these participants were also omitted.

For each participant, we calculated the mean RT for correct responses as well as the accuracy rate for problems in either condition.

Since our stimuli sets contained a difference in SDN size between problems with a small SDN with or without carryover, a repeated-measures ANOVA with two within-subjects variables (small/large SDN and carry/no-carry status) was not advisable. Nevertheless, we present such an analysis^3^, which shows main effects of SDN size and carry status on RT and on accuracy. A repeated-measures ANOVA with the sets of noncarry problems showed that participants solved problems with a small SDN faster than problems with a large SDN, *F*(1, 28) = 59.01,* p* <.001, *MSE* = 119,483.24, partial η^2^ =.678. A repeated-measures ANOVA with the carry problems showed that participants solved problems with a small SDN faster than problems with a large SDN, *F*(1, 28) = 43.54, *p* <.001, *MSE* = 184,537.98, partial η^2^ =.609. A repeated-measures ANOVA with the sets of noncarry problems showed no difference in accuracy whether participants solved problems with a small or a large SDN size, *F*(1, 28) =.18, *p* =.668, *MSE* = 17.66, partial η^2^ =.007. A repeated-measures ANOVA with the carry problems showed that participants solved small SDN problems more accurately than large SDN problems, *F*(1, 28) = 4.32, *p* =.047, *MSE =* 27.69, partial η^2^ =.134 (see Table 2).

**Table 2:** Mean RT and accuracy rates for problems with small and large SDN, with and without carry in Experiment 3

		Small SDN		Large SDN		
		Mean	*SD*	Mean	*SD*	*p*
RT	Without carry	3,892.98	1,113.24	4,590.28	1,071.33	.000
	With carry	5,204.19	1,498.11	5,948.59	1,463.96	.000
Acc	Without carry	94.64	5.08	94.16	5.72	.668
	With carry	88.12	7.34	85.25	8.97	.047

#### The use of strategies

First, we calculated the rate of strategy use by problem type across all participants (see Table S4 in the Supplementary Material). The use of retrieval indicates a strong association between the operand pair and the solution (Siegler, 1988), which may lead to stronger activation of the problem’s SDN and to shorter RTs relative to the use of calculation strategies. All strategies except Strategy 1 (retrieval) involved calculation. Thus, we compared between retrieval and all other strategies. For each participant we calculated the percentage of using retrieval to solve problems under each condition.^4^ A repeated-measures ANOVA with the noncarry problem sets showed that problems with a small SDN were solved using retrieval more than problems with a large SDN size, *F*(1, 28) = 16.61, *p* <.001, *MSE* = 373.56, partial η^2^ =.372. A repeated-measures ANOVA with the carry problems showed that participants made more use of retrieval when solving problems with a small SDN than with a large SDN, *F*(1, 28) = 9.50, *p* =.005, *MSE* = 85.18, partial η^2^ =.253 (see Table 3).

**Table 3:** Mean rates for using retrieval to solve problems with small and large SDN, with and without carry in Experiment 3

	Small SDN		Large SDN		
	Mean	*SD*	Mean	*SD*	*p*
Without carry	44.83	34.82	24.14	25.43	.000
With carry	13.22	16.28	5.75	12.01	.005

For each participant in each condition, we calculated the average RT for problems that this participant reported to have solved by retrieval and for problems that this participant reported to have solved by other strategies. As Table 3 shows, the use of retrieval varied widely between conditions, and was very low for problems with carry and a large SDN. Thus, we analyzed the data for problems with and without carryover together. A repeated-measures ANOVA with two within-subjects variables (small/large SDN, retrieval/calculation) showed a main effect of SDN size on RT. Participants solved problems with a small SDN more quickly than problems with a large SDN, *F*(1, 20) = 26.38, *p* <.001, *MSE* = 319493.55, partial η^2^ =.569. There was a main effect of strategy on RT. Participants solved problems with retrieval more quickly than with calculation*, F*(1, 20) = 38.68, *p* <.001, *MSE* = 662,542.27, partial η^2^ =.659 (see Table 4). There was no interaction between SDN size and strategy on RT, *F*(1, 20) =.193, *p* =.665, *MSE* = 294,514.62, partial η^2^ =.010. Most importantly, the SDN effect occurred regardless of strategy. When using retrieval, participants solved problems with a small SDN more quickly than problems with a large SDN, *F*(1, 20) = 7.82, *p* =.011, *MSE* = 454076.61, partial η^2^ =.281. This pattern repeated itself for the use of calculation. When using calculation, participants solved problems with a small SDN more quickly than problems with a large SDN, *F*(1, 20) = 30.86, *p* <.001, *MSE* = 159931.56, partial η^2^ =.607.

**Table 4:** Mean RT for participants using retrieval versus calculation to solve problems with small and large SDN in Experiment 3

	Small SDN		Large SDN		
	Mean	*SD*	Mean	*SD*	*p*
Retrieval	3,426.47	1,090.55	4,007.95	1,239.65	.011
Calculation	4,479.09	1,353.81	5,164.65	1,214.69	.000

### Discussion

In Experiment 3, we presented participants with the same sets as in Experiment 2 and added a strategy questionnaire. In line with the results of Experiment 2, the results of the current experiment showed that participants solved noncarry problems with a small SDN faster than noncarry problems with a large SDN. There was no difference in accuracy for problems with a small or large SDN with no carryover. Participants solved carryover problems with a small SDN faster and more accurately than carryover problems with a large SDN.

Participants used retrieval more when solving complex problems without carryover than complex problems with carryover. Participants used retrieval more when solving noncarry problems with a small SDN than noncarry problems with a large SDN. This pattern repeated itself for problems with carryover: participants used retrieval more when solving carryover problems with a small SDN than carryover problems with a large SDN. In other words, regardless of carryover status, participants used retrieval more when solving problems with a small SDN than when solving problems with a large SDN.

In line with previous research (Lemaire & Siegler, 1995; Siegler, 1988; Tronsky, 2005), when using retrieval, participants solved problems more quickly than when using calculation. Most importantly, when using retrieval, participants solved problems with a small SDN more quickly than problems with a large SDN. This pattern repeated itself for the use of calculation: When using calculation, participants solved problems with a small SDN more quickly than problems with a large SDN.

In Experiment 2, participants solved the problems in all four conditions with equally high accuracy rates. In Experiment 3, there was no difference in accuracy between noncarry problems with a small and a large SDN. However, carry problems with a small SDN were solved more accurately than carry problems with a large SDN. The carryover problems in Experiment 3 were solved with lower degrees of accuracy than the carryover problems in Experiment 2, allowing for these differences to occur. Since carry problems are solved more frequently by calculation (Tronsky, 2005), and since calculation is more prone to errors than retrieval (Siegler, 1988; Tronsky, 2005), accuracy differences are more likely to occur in problems with carryover. In line with our previous findings with single-digit problems (Sapir-Yogev et al., 2024), the current study shows that problems in the SDN interfere with the solution of a complex problem. The larger the SDN, the larger the interference. In the current Experiment, carryover problems with a small SDN may have been solved more accurately than carryover problems with a large SDN because they were subjected to less interference due to the smaller number of problems in their SDNs.

Regardless of carry status, problems with a small SDN were solved more often using a retrieval strategy than problems with a large SDN. Since the solution of problems with a small SDN is subjected to less interference than the solution of problems with a large SDN, there is a greater chance to arrive at the correct solution to small-SDN problems whether they are retrieved or calculated. Since problems with a smaller SDN receive less interference, and are more likely to be solved correctly, solving them correctly several times facilitates the formation of holistic representations of these problems in memory, and increases the use of retrieval (Siegler, 1988).

## General discussion

In this study, we planned to explore automatic activation of the Shared-Digit Network (SDN) in the context of complex multiplication problems. We defined the SDN of a complex problem as composed of all *single-digit* problems sharing digits with this complex problem. We argued that all digits in a complex problem automatically activate all single-digit problems sharing them, and these activated problems then interfere with the solution of the target complex problem. The larger a problem’s SDN, the greater its interference, and the slower and less accurate the complex problem’s solution. In three experiments we have demonstrated that SDN size determines performance in the solution of complex multiplication problems.

Experiment 1 showed that SDN size predicted speed and accuracy beyond problem size in all complex multiplication problems with solutions that are smaller than 100. Experiment 2 showed a similar pattern with English-speaking participants, solving four sets of carry and noncarry problems matched in problem size but differing in SDN size. Regardless of carryover status, participants solved problems with a small SDN faster than problems with a large SDN. Experiment 3 replicated the findings of Experiment 2, by showing that regardless of carryover status, participants solved problems with a small SDN faster than problems with a large SDN. Moreover, Experiment 3 showed that regardless of carryover status, participants used retrieval more often when solving problems with a small SDN than with a large SDN. Finally, whether they used retrieval or calculation, participants solved problems with a small SDN more quickly than problems with a large SDN.

Together, the results of these three experiments provide evidence that the SDN size of a complex problem determines speed in its solution. These findings apply to problems solved by retrieval as well as to problems solved by calculation. They apply to no-carry problems as well as to carry problems. We argue that the problems that constitute SDN, and share digits with the target problem, interfere with its solution. The smaller the SDN, the more quickly the problem is solved, because fewer problems interfere with its solution.

Previous studies have shown an automatic activation of multiplication problems, even in tasks that do not require multiplication (Galfano et al., 2009; Keha et al., 2024; Winkelman & Schmidt, 1974). Although the task used in the current study did require the use of multiplication, it involved the solution of complex rather than single-digit multiplication problems. Nevertheless, single-digit problems, most of which were irrelevant to the task, were automatically activated during the performance of this task. This finding extends our previous finding (Sapir-Yogev et al., 2024) in that the automatic activation of SDN occurs not only in the solution of single-digit problems, which compose the SDN itself, but also in complex problems, which do not compose the SDN.

Our results replicate some of the major effects of multiplication with complex problems. Experiment 1 attests to the existence of the problem-size effect in complex multiplication, as found in previous studies (Lemaire & Brun, 2016; Liu, 2013; van der Ven et al., 2015). Results of Experiment 1 also show the five effect. We argue that complex five problems are solved more quickly than complex nonfive problems, because they have smaller SDNs. Experiments 2 and 3 replicated the finding that participants solve noncarry problems more quickly and more accurately than carry problems (Tronsky, 2005). In the set of complex problems whose solutions are smaller than 100, noncarry problems have smaller SDNs than carry problems (see Appendix 1), and are thus exposed to less interference. This may be one of the reasons that noncarry problems are easier to solve versus carry problems (Imbo & LeFevre, 2010). Previous studies have found that participants solved single-digit problems by retrieval more quickly than by calculation (e.g., Lemaire & Siegler, 1995; Shrager & Siegler, 1998; Siegler & Shipley, 1995). Experiment 3 replicated these findings and extended them to complex problems (see also Tronsky, 2005). Due to the scarcity of research with complex multiplication (Liu, 2013; van der Ven et al., 2015), these findings are important in themselves.

Complex multiplication problems, especially those with carryover, are solved with a variety of strategies (Hickendorff et al., 2019; Tronsky, 2005, 2016). Our results show that whether participants use retrieval or calculation strategies, problems with smaller SDNs are solved faster than problems with larger SDNs. The single-digit problems composing the SDN are automatically activated by all digits in the complex problem, in the operands and in the solution. How do the digits in the solution of a complex problem activate the single digit problems sharing them? Some complex problems that were presented in our experiments (e.g., 2 × 12 =, 3 × 25 =) may have been solved by retrieval (Tronsky, 2016). In these problems, presentation of the operands may have activated the entire problem (Campbell & Oliphant, 1992). Other complex problems that we presented may have been solved mostly by calculation, but even these problems had probably been practiced in childhood (Heirdsfield et al., 1999; LeFevre et al., 1993; Liu, 2013) and many of their solutions may have been familiar. The SDN effect in calculated problems may have been facilitated by a horse-race pattern (Ashcraft, 1987), during which the solution may have been retrieved after partial computation (Tronsky, 2005). Further research is needed to examine these suggested explanations.

Calculated and retrieved problems may be solved via different routes, which may be simultaneously activated. The triple-code model (Dehaene, 1992; Dehaene & Cohen, 1995) suggests direct and indirect routes for solving multiplication problems. In the direct route, the problems are visually identified and then transcoded into a verbal, asemantic representation of the problem (e.g., “two times four, eight”). This route may be used to retrieve the solutions of holistically represented problems. The indirect route is used when the problem is relatively unknown, as may be the case for complex problems that are solved by calculation. The operands are encoded as quantity representations and then semantically manipulated to calculate the solution (Dehaene & Cohen, 1997). Dehaene and Cohen (1997) suggest that arithmetic operations may involve the simultaneous activation of both the direct and indirect routes, a process resembling a horse-race pattern. Siegler and Shipley (1995) suggested that participants choose to use retrieval or calculation strategies on the basis of gathered information, such as speed of execution and precision in past attempts. Repeated use of calculation leads to a stronger representation of the problem in memory and to an increased likelihood of its retrieval in future attempts. It is possible that a small SDN leads to a faster shift from calculation to retrieval strategies relative to a large SDN, because when a problem has a small SDN, fewer problems interfere with its solution.

In Experiment 1, we showed that an SDN based on the operands of a complex problem and an SDN based on the digits in the solution of a complex problem predict unique variance in RT and in accuracy. It may be interesting to create balanced sets of complex problems with a large versus small SDN-by-operands or SDN-by-solution, to further investigate this phenomenon. Another interesting question is whether an SDN that includes the digits of the intermediate problems that are used during the calculation of a complex problem would also predict performance.

Our results show that the SDN effect occurs for problems that are retrieved as well as calculated, and regardless of carryover status. However, we chose a relatively easy set of complex problems, which were solved with high accuracy rates. It is not clear whether complex problems with more different digits, such as 12 × 38 = 456, would display the SDN effect. Such problems may require much greater working memory resources to execute, and a more complex algorithm (Imbo, et al., 2007a, Imbo et al., 2007b; Tronsky, 2005). These factors may influence performance more than the SDN.

In this study, we chose all complex problems with solutions that are smaller than 100. We could have chosen a larger or a smaller set of complex problems. It has yet to be investigated whether the SDN effect applies to all such sets. Moreover, we defined the SDN of a complex problem as the number of single-digit problems sharing at least one digit with the target problem. However, complex multiplication problems may form a network between them and affect each other (Tronsky; 2016). The SDN of a complex multiplication problem may be composed of both single-digit and complex problems sharing at least one digit with it. In this study, we were interested in the automatic activation of the well-rehearsed single-digit problems, and thus did not include complex problems in the SDN. Moreover, boundaries around the set of complex problems are arbitrarily defined, and it is unclear which complex problems should be included in such an SDN.

## Limitations

We acknowledge that our experiments may have some limitations. In all three experiments, we presented the problems with the single-digit operand first. Tronsky (2016) found no evidence of an operand order effect in complex multiplication. He let participants practice complex problems with the larger or the smaller operand first. In a number-matching task, participants were slower to respond when the third presented number was the product of the first two presented numbers, than when it was not the product of the first two, and this effect was the same when the two cues were presented in their practiced or unpracticed operand order. Nevertheless, it would be interesting to see whether the SDN effect occurs when the problems are presented with the double-digit operand first.

We used a permissive time limit in order to reduce anxiety, not to reduce accuracy, to give participants sufficient time to respond and thus not limit the range of RT in the solution of various problems. Permissive or no time limits have been used in other complex multiplication studies (e.g., Liu, 2013). It is possible, however, that using a shorter time limit would have led to decreased accuracy levels (Koscinski & Gast, 1993; Standage et al., 2014), which would have enabled us to obtain more accuracy effects. Our selected set of complex multiplication problems has somewhat artificial boundaries. However, our definition of the SDN, based on the single-digit network, makes it possible to explore these phenomena in any chosen set. In Experiment 3, we inquired about strategies after the participants solved all problems. Since the use of strategies was not one of our main hypotheses, we preferred to assess them in a way that avoids introducing introspection that could affect RT. Future experiments which focus on the effect of SDN on strategy selection should inquire about strategies after the solution of each problem.

In Experiment 1 we showed the SDN effect explains variance in speed and accuracy beyond problem size. In Experiments 2 and 3 we did not include problems with the operands five, ten, and 11, which may be solved by rule. The complex problem sets in these two Experiments were also matched in problem size, in the single-digit operands, and in carryover status. Nevertheless, other factors that were not explored in this study may correlate with SDN size. For example, working memory may affect problems with a large SDN more than problems with a small SDN. Carryover problems involve more working memory resources than noncarry problems (Imbo et al., 2007a, Imbo et al., 2007b). However, the SDN effect appeared in both problem types. The relations between working memory and SDN size should be explored in future studies, along with other potential alternative explanations to the SDN effect.

## Conclusion

This is the first study to delineate an automatic activation of well-defined networks of single-digit problems that affect the solution of complex multiplication problems. The number of single-digit multiplication problems that share at least one digit with the digits in a complex multiplication problem, or its SDN size, presents a parsimonious explanation to variance in speed and accuracy in the solution of complex multiplication problems, and to the five-effect in complex multiplication.

## Electronic supplementary material

Below is the link to the electronic supplementary material.

Supplementary file1 (DOCX 65 KB)

## Data Availability

The data for all Experiments in this article are publicly available on the Open Science Framework. (https://osf.io/jnh76/?view_only=893584d08e524cf4a2075078ab8691e8).

## Appendix 1 Multiplication problems, number of different digits, network size, and problem size

**Table:**

	Problem	Problem size	Number of different digits	SDN size	Carryover
1	2 × 10 =	20	3	25	0
2	2 × 11 =	22	2	23	0
3	2 × 12 =	24	3	30	0
4	2 × 13 =	26	4	33	0
5	2 × 14 =	28	4	31	0
6	2 × 15 =	30	5	33	1
7	2 × 16 =	32	4	33	1
8	2 × 17 =	34	5	36	1
9	2 × 18 =	36	5	34	1
10	2 × 19 =	38	5	36	1
11	2 × 20 =	40	3	28	0
12	2 × 21 =	42	3	30	0
13	2 × 22 =	44	2	27	0
14	2 × 23 =	46	4	35	0
15	2 × 24 =	48	3	30	0
16	2 × 25 =	50	3	26	1
17	2 × 26 =	52	3	33	1
18	2 × 27 =	54	4	32	1
19	2 × 28 =	56	4	34	1
20	2 × 29 =	58	4	34	1
21	2 × 30 =	60	4	33	0
22	2 × 31 =	62	4	33	0
23	2 × 32 =	64	4	35	0
24	2 × 33 =	66	3	32	0
25	2 × 34 =	68	5	36	0
26	2 × 35 =	70	5	32	1
27	2 × 36 =	72	4	33	1
28	2 × 37 =	74	4	35	1
29	2 × 38 =	76	5	35	1
30	2 × 39 =	78	5	35	1
31	2 × 40 =	80	4	31	0
32	2 × 41 =	82	4	31	0
33	2 × 42 =	84	3	30	0
34	2 × 43 =	86	5	36	0
35	2 × 44 =	88	3	30	0
36	2 × 45 =	90	5	34	1
37	2 × 46 =	92	4	34	1
38	2 × 47 =	94	4	32	1
39	2 × 48 =	96	5	34	1
40	2 × 49 =	98	4	32	1
41	3 × 10 =	30	3	23	0
42	3 × 11 =	33	2	21	0
43	3 × 12 =	36	4	33	0
44	3 × 13 =	39	3	25	0
45	3 × 14 =	42	4	35	1
46	3 × 15 =	45	4	35	1
47	3 × 16 =	48	5	35	1
48	3 × 17 =	51	4	31	1
49	3 × 18 =	54	5	36	1
50	3 × 19 =	57	5	31	1
51	3 × 20 =	60	4	33	0
52	3 × 21 =	63	4	33	0
53	3 × 22 =	66	3	32	0
54	3 × 23 =	69	4	35	0
55	3 × 24 =	72	4	35	1
56	3 × 25 =	75	4	32	1
57	3 × 26 =	78	5	35	1
58	3 × 27 =	81	5	34	1
59	3 × 28 =	84	4	36	1
60	3 × 29 =	87	5	35	1
61	3 × 30 =	90	3	23	0
62	3 × 31 =	93	3	25	0
63	3 × 32 =	96	4	35	0
64	3 × 33 =	99	2	20	0
65	4 × 10 =	40	3	27	0
66	4 × 11 =	44	2	26	0
67	4 × 12 =	48	4	31	0
68	4 × 13 =	52	5	36	1
69	4 × 14 =	56	4	33	1
70	4 × 15 =	60	5	33	1
71	4 × 16 =	64	3	31	1
72	4 × 17 =	68	5	34	1
73	4 × 18 =	72	5	33	1
74	4 × 19 =	76	5	35	1
75	4 × 20 =	80	4	31	0
76	4 × 21 =	84	4	31	0
77	4 × 22 =	88	3	30	0
78	4 × 23 =	92	4	35	1
79	4 × 24 =	96	4	34	1
80	5 × 10 =	50	3	19	0
81	5 × 11 =	55	2	19	0
82	5 × 12 =	60	5	34	1
83	5 × 13 =	65	4	31	1
84	5 × 14 =	70	5	33	1
85	5 × 15 =	75	3	25	1
86	5 × 16 =	80	5	32	1
87	5 × 17 =	85	4	30	1
88	5 × 18 =	90	5	31	1
89	5 × 19 =	95	3	25	1
90	6 × 10 =	60	3	24	0
91	6 × 11 =	66	2	22	0
92	6 × 12 =	72	4	33	1
93	6 × 13 =	78	5	32	1
94	6 × 14 =	84	4	32	1
95	6 × 15 =	90	5	31	1
96	6 × 16 =	96	3	27	1
97	7 × 10 =	70	3	22	0
98	7 × 11 =	77	2	19	0
99	7 × 12 =	84	5	33	1
100	7 × 13 =	91	4	28	1
101	7 × 14 =	98	5	32	1
102	8 × 10 =	80	3	22	0
103	8 × 11 =	88	2	20	0
104	8 × 12 =	96	5	35	1
105	9 × 10 =	90	3	22	0
106	9 × 11 =	99	2	19	0
With	Mean	70.67	4.40	32.84	
carryover	*SD*	19.04	0.67	2.56	
Without	Mean	61.94	3.21	27.94	
carryover	*SD*	22.82	0.81	5.31	

*Note.* A univariate ANOVA showed that no-carry problems have a smaller SDN than carry problems, *F*(1, 104) = 37.93, *p* <.001, *MSE* = 16.67, partial η^2^ =.267

## Appendix 2 Stimuli in Experiments 2 and 3

**Table:**

Small SDN without carry			Large SDN without carry		
Problem	Problem size	SDN size	Problem	Problem size	SDN size
2 × 12 =	24	30	2 × 13 =	26	33
2 × 21 =	42	30	2 × 23 =	46	35
2 × 24 =	48	30	2 × 32 =	64	35
2 × 42 =	84	30	2 × 34 =	68	36
3 × 13 =	39	25	3 × 21 =	63	33
3 × 31 =	93	25	3 × 23 =	69	35
Mean *SD*	55.00 24.9	28.33 2.36	Mean *SD*	56.00 15.41	34.50 1.11
Small SDN with carry			Large SDN with carry		
Problem	Problem size	SDN size	Problem	Problem size	SDN size
2 × 16 =	32	33	2 × 17 =	34	36
2 × 27 =	54	32	2 × 19 =	38	36
2 × 36 =	72	33	2 × 38 =	76	35
2 × 49 =	98	32	2 × 39 =	78	35
3 × 17 =	51	31	3 × 18 =	54	36
3 × 19 =	57	31	3 × 26 =	78	35
Mean *SD*	60.67 20.39	32.00 .81	Mean *SD*	59.67 18.70	35.50 .50

## Footnotes

1 Many of the noncarry problems with a small SDN are five, ten or 11 problems, making it impossible to select sets in which the small SDN sizes are smaller than the median SDN size. Thus, we chose six noncarry problems with the smallest possible SDN, and six noncarry problems with the largest possible SDN, matching in single-digit operands and problem size. We used the same method to choose the sets of carry problems. Independent-samples *t* tests revealed no difference in problem size between problems with or without carryover, *t*(22) = −.543, *SE* = 8.59, *p =*.593, no difference in problem size between problems with a large or a small SDN, *t*(22) = −.000, *SE* = 1.00, *p =* 1.00, and no difference in SDN between problems with or without carryover, *t*(22) = −1.91, *SE* = 1.22, *p =*.069. However, there was a significant difference in SDN size between problems having a small SDN size with or without carry, *t*(10) = −3.28, *SE* = 1.11, *p =*.008. In general, problems with carryover have larger SDN sizes than problems without carryover, *t*(104) = −6.21, *SE* =.79, *p <*.000. We could not match the small SDN sizes of problems with and without carryover, because it was not possible to create a set of problems with carryover, matching in the single digit operands and in problem size, that has a smaller SDN size than the one we created.

2 A repeated-measures ANOVA with two within-subjects variables (SDN size and carry/no-carry status) showed a main effect of SDN size *F*(1, 29) = 27.62, *p* <.001, *MSE* = 145524.903, partial η^2^ =.49 and a main effect of carry status *F*(1, 29) = 17.42,, *p* <.001, *MSE* = 252611.714, partial η^2^ =.37. There was no interaction between SDN size and carry status, *F*(1, 29) = 1.48, *p* =.234, *MSE* = 63067.623, partial η^2^ =.05.

3 A repeated-measures ANOVA with two within-subjects variables (SDN size and carry/no-carry status) showed a main effect of SDN size on RT, *F*(1, 28) = 83.79, *p* <.001, *MSE* = 179831.37, partial η^2^ =.750 and a main effect of carry status on RT *F*(1, 28) = 57.88, *p* <.001, *MSE* = 892677.49, partial η^2^ =.674. There was no interaction between SDN size and carry status on RT, *F*(1, 28) =.129, p =.722, *MSE* = 124189.85, partial η^2^ =.005. A repeated-measures ANOVA with two within-subjects variables (SDN size and carry/no-carry status) showed a main effect of SDN size on accuracy, *F*(1, 28) = 4.27, *p* =.048, *MSE* = 19.067, partial η^2^ =.132 and a main effect of carry status on accuracy, *F*(1, 28) = 30.77, *p* <.001, *MSE* = 56.02, partial η^2^ =.524. There was no interaction between SDN size and carry status on accuracy, *F*(1, 28) = 1.58, *p* =.219, *MSE* = 26.28, partial η^2^ =.053.

4 A repeated-measures ANOVA with two within-subjects variables (SDN size and carry/no-carry status) showed a main effect of SDN size on strategy use, *F*(1, 28) = 19.61, *p* <.001, *MSE* = 293.17, partial η^2^ =.412, and a main effect of carry status on strategy use, *F*(1, 28) = 33.83, *p* <.001, *MSE* = 535.71, partial η^2^ =.547. There was an interaction between SDN size and carry status on strategy use, *F*(1, 28) = 7.65, *p* =.010, *MSE* = 165.57, partial η^2^ = 215.
