# Supplementary material for: Automatic activation of the shared-digit network in the solution of complex multiplication problems
Source: Mem Cognit. 2025 Aug 15;54(2):532–47. doi: 10.3758/s13421-025-01766-1 (PMC12957635; doi:10.3758/s13421-025-01766-1)
Supplement: Supplementary file 1 — Supplementary file1 (DOCX 65 KB) [file 13421_2025_1766_MOESM1_ESM.docx]

**Supplementary Material**

**Figure S1**

*Sequence of Screen Presentations in Experiments 1, 2 and 3*

**
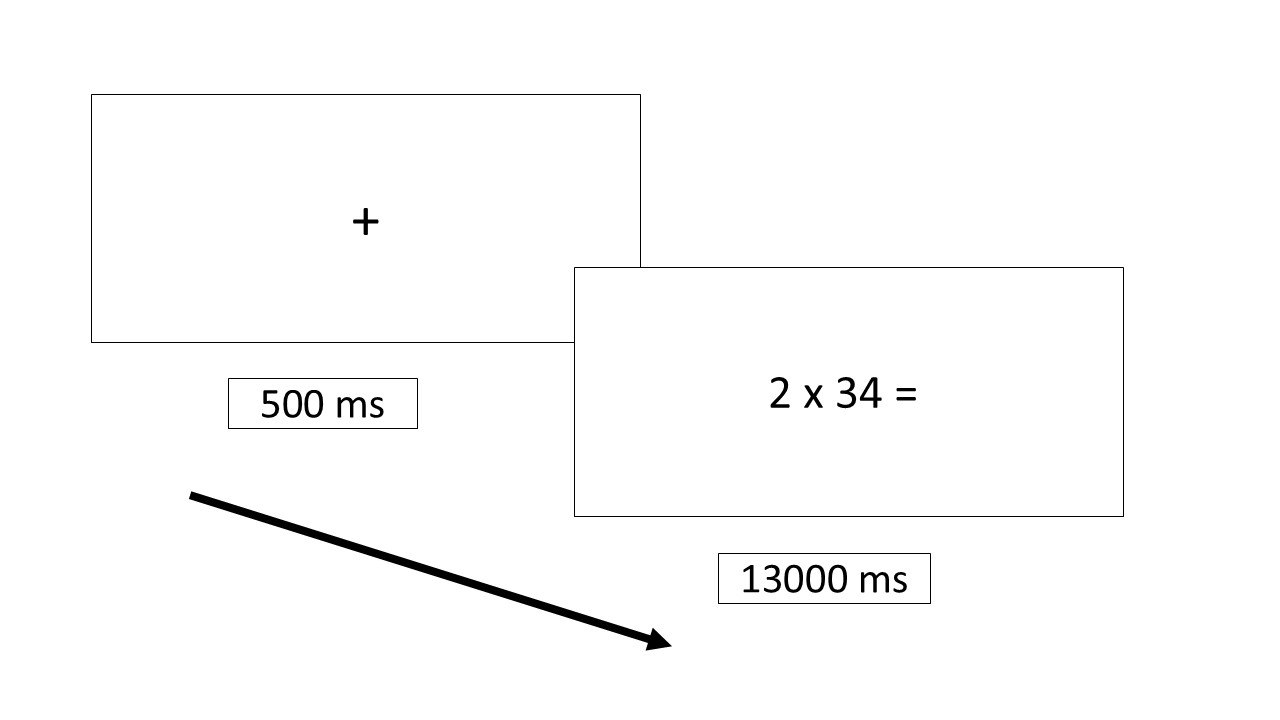
**

**Table S1**

*Linear Regressions Predicting RT and Accuracy Rate in Experiment 1 including all problems except multiples of 10 and 11*

| Dep. var | Step | Indep. var | B | SE | *β* | t | p | ∆*R*2 |
| --- | --- | --- | --- | --- | --- | --- | --- | --- |
| RT | Step 1 | Problem size | 18.85 | 6.85 | .28 | 2.75 | .007 | .079 |
|  | Step 2 | Problem size | 22.45 | 6.44 | .33 | 3.49 | .001 |  |
|  |  | SDN size | 162.35 | 41.70 | .37 | 3.89 | .000 | .137 |
| Acc | Step 1 | Problem size | -.18 | .05 | -.38 | -3.80 | .000 | .141 |
|  | Step 2 | Problem size | -.19 | .05 | -.41 | -4.19 | .000 |  |
|  |  | SDN size | -.68 | .30 | -.22 | -2.31 | .023 | .049 |

**Table S2**

*Linear Regressions Predicting RT and Accuracy Rate in Experiment 1 with All Complex Problems, with the Predictors SDN-by-Operands and SDN-by-Solution*

| Dep. var | Step | Indep. var | B | SE | *β* | t | p | ∆*R*2 |
| --- | --- | --- | --- | --- | --- | --- | --- | --- |
| RT | Step 1 | Problem size | 22.35 | 6.81 | .31 | 3.28 | .001 | .094 |
|  | Step 2 | Problem size | 19.79 | 5.92 | .27 | 3.34 | .001 |  |
|  |  | SDN  operands^^[[1]](#footnote-1)^^ | 182.29 | 30.72 | .48 | 5.93 | .000 | .231 |
|  | Step 3 | Problem size | 29.66 | 5.71 | .41 | 5.19 | .000 |  |
|  |  | SDN  operands | 85.23 | 34.02 | .22 | 2.50 | .014 |  |
|  |  | SDN solution^^[[2]](#footnote-2)^^ | 150.74 | 30.58 | .46 | 4.93 | .000 | .130 |
| Acc | Step 1 | Problem size | -.17 | .04 | -.38 | -4.18 | .000 | .144 |
|  | Step 2 | Problem size | -.16 | .04 | -.36 | -4.13 | .000 |  |
|  |  | SDN operands | -.69 | .20 | -.30 | -3.41 | .001 | .087 |
|  | Step 3 | Problem size | -.19 | .04 | -.42 | -4.63 | .000 |  |
|  |  | SDN operands | -.40 | .24 | -.17 | -1.65 | .102 |  |
|  |  | SDN solution | -.44 | .22 | -.22 | -2.03 | .045 | .030 |

|  |
| --- |
|  |
|  |
|  |
|  |

**Table S3**

*Response Time and Accuracy by Five and Non-Five Problems in Experiment 1*

|  | Problem  Size | | SDN  size | | RT | | Accuracy  (percent correct) | |
| --- | --- | --- | --- | --- | --- | --- | --- | --- |
|  | Mean | SD | Mean | SD | Mean | SD | Mean | SD |
| Five problems | 70 | 17.52 | 30.73 | 3.21 | 3722.91 | 945.61 | 92.52 | 6.16 |
| Non-five problems | 67.76 | 21.09 | 32.57 | 2.97 | 4688.67 | 1124.25 | 89.42 | 5.16 |

**Supplemental Analyses of the Five effect in Experiment 1 data**

To look for the five effect, we calculated the mean RT and the mean accuracy rate for five and non-five problems for each participant. Five problems were defined as all problems with at least one operand that is equal to five or is a multiple of five. Non-five problems were defined as all problems with no operands that are equal to five or multiples of five. The problems in this analysis did not include problems with the operands 10 or 11.

Repeated ANOVA measures showed that participants solved five-problems more quickly, F (1, 30) = 112.37, MSE = 128653.42, p < .001, partial η2 = .789, and more accurately, F (1, 30) = 6.09, MSE = 24.44, p = .019, partial η2 = .169, than non-five problems.

**Table S4**

*Rate of Strategy Use by Problem Type Across All Participants*

|  | Strategy | | | | | |
| --- | --- | --- | --- | --- | --- | --- |
| Problem Type | 1  Retrieval | 2  Calculation | 3  Rounding | 4  Familiar Problem | 5  Algorithm | 6  Addition |
| Small SDN no Carry | 44.83 | 34.48 | 8.04 | .57 | 1.72 | 10.34 |
| Large SDN no Carry | 24.14 | 55.75 | 4.03 | 1.72 | 4.02 | 10.34 |
| Small SDN with Carry | 13.22 | 50 | 24.71 | 1.15 | 3.45 | 7.47 |
| Large SDN with Carry | 5.75 | 51.15 | 26.44 | 2.87 | 6.89 | 6.89 |

| Note: Strategy 1: Remembered/knew the solution immediately, with no need for any calculation. Strategy 2: Multiplied the tens digit, then multiplied the units digit, and then added or the other way around. For example: 4 x 19 = (4 x 10) + (4 x 9) or 4 x 19 = (4 x 9) + (4 x 10). Strategy 3: Rounded the double-digit number up or down and then subtracted or added the difference. For example: 4 x 19 = (4 x 20) – 4 or 4 x 21 = (4 x 20) + 4. Strategy 4: Relied on familiar problems. For example: 4 x 19 = (4 x 15) + (4 x 4). Strategy 5: Mentally carried out the standard multidigit multiplication algorithm. Strategy 6: Turned the problem into an addition problem. For example: 2 x 11 = 11 + 11. |
| --- |

1. SDN operands: the number of single digit problems sharing at least one digit with the digits in the operands. [↑](#footnote-ref-1)
2. SDN solution: the number of single digit problems sharing at least one digit with the digits in the solution. [↑](#footnote-ref-2)
